# An Enhanced Method for Transmitochondrial Cybrid Generation

**DOI:** 10.3390/cells15100898

**Published:** 2026-05-14

**Authors:** Luke Weaver, Mikhail F. Alexeyev

**Affiliations:** 1 Department of Physiology and Cell Biology, University of South Alabama, Mobile, AL 36688, USA; lw2433@jagmail.southalabama.edu; 2 Department of Biomedical Sciences, University of South Alabama, Mobile, AL 36688, USA

**Keywords:** mitochondrial DNA, chemical enucleation, mitomycin C, niclosamide, carbonyl cyanide m-chlorophenylhydrazone

## Abstract

Transmitochondrial cybrid technology is a key approach for elucidating the effects of mitochondrial DNA (mtDNA) mutations in defined nuclear genetic backgrounds and for studying nuclear–mitochondrial interactions. However, its application is limited by the availability of suitable recipient cell lines and by technically demanding enucleation procedures. We report three advances in cybrid technology: (1) enucleation using mitomycin C, a widely used agent for generating feeder layers in stem cell culture, which does not depend on cell attachment and provides a gentler alternative to actinomycin D; (2) selection of cybrids using mitochondrial uncouplers, which can reduce background survival of non-cybrid cells; and (3) cryopreservation of enucleated donor cells in liquid nitrogen, preserving fusion competence and increasing experimental flexibility. Additionally, we validate newly developed mtDNA-free (ρ^0^) derivatives of HCT116, HT1080, and U2OS cell lines as recipients for cybrid generation. These advances facilitated donor cell preparation, improved cybrid selection, and enhanced experimental flexibility, including the demonstration of preserved fusion competence of enucleated HeLa cells after 10 years of cryostorage. The ρ^0^ derivatives of HCT116, HT1080, and U2OS cells were confirmed as effective recipients. Together, these improvements enhance the efficiency and accessibility of transmitochondrial cybrid technology and are expected to facilitate its broader application.

## 1. Introduction

Mitochondria are widely accepted to have originated from an ancestral prokaryotic organism that was engulfed by a precursor eukaryotic cell through endosymbiosis [1]. In this evolutionary framework, mitochondrial DNA (mtDNA) is regarded as a remnant of the genome of this ancestral symbiont. The human mitochondrial genome encodes 13 polypeptides that are essential components of oxidative phosphorylation (OXPHOS) complexes I, III, IV, and V, as well as the full complement of transfer RNAs (tRNAs) and ribosomal RNAs (rRNAs) required for their translation within the organelle [2]. However, the vast majority of mitochondrial proteins are encoded by nuclear DNA (nDNA). These include the remaining 36 subunits of complex I, 10 subunits of complex III, 10 subunits of complex IV, and 14 subunits of complex V, along with approximately 1500 additional proteins that comprise the mitochondrial proteome [3,4].

Since the late 1980s, when mutations in mtDNA were first linked to severe and often fatal human diseases, there has been growing recognition of the central role of mitochondrial dysfunction in human pathology [5,6,7]. Alterations in mtDNA have now been implicated in disorders affecting virtually all organ systems, including the skeletal [8,9], muscular [10], respiratory [11,12], digestive and excretory [13,14], urinary [15], circulatory [16,17], integumentary [18,19], endocrine [10], nervous [20,21], lymphatic [22,23], and reproductive systems [24,25,26]. This broad involvement underscores the fundamental importance of mtDNA in cellular physiology and has driven sustained interest in understanding its role in health and disease.

Despite this interest, dissecting the specific contributions of mtDNA to cellular function and pathology remains challenging due to the dual genetic origin of the mitochondrial proteome [27,28]. Mitochondrial function depends on a coordinated expression of both nuclear and mitochondrial genomes, and mutations in either genome, or interactions between them, can lead to dysfunction. Notably, identical mtDNA mutations can produce different phenotypic outcomes depending on the nuclear genetic background, highlighting the importance of nucleo-mitochondrial interactions [27,29,30]. To address this complexity, experimental systems that allow the study of mtDNA variants within a controlled nuclear background have been developed. One such system is the transmitochondrial cybrid (cytoplasmic hybrid), generated by fusing enucleated donor cells (cytoplasts) with recipient cells lacking mtDNA (ρ^0^ cells) [31,32]. This approach, first described by King and Attardi in 1989, has been instrumental in elucidating the functional consequences of mtDNA mutations [31]. Studies using cybrid models have characterized mutations within the mitochondrial genome responsible for pathologies such as cancer progression, age-related macular degeneration, neurodegenerative disorders, mitochondrial encephalomyopathy, vascular diseases, and developmental delays [29,30,33,34,35,36,37,38] (for reviews, see [39,40,41,42]). However, the limited repertoire of the available ρ^0^ cell lines has limited the utility of the approach. Previously, we addressed this issue by developing several techniques for inducing mtDNA loss in cultured cell lines [43,44,45,46].

The requirement for mechanical enucleation of donor cells remains a significant limitation, particularly for cells that grow in suspension or adhere poorly to substrates [31,32]. In 2003, a modification of the King and Attardi’s replaced mechanical enucleation with chemical enucleation using the antineoplastic antibiotic actinomycin D (ActD) [32]. ActD intercalates into double-stranded DNA at GpC sites within the minor groove via its phenoxazone ring, thereby inhibiting DNA replication and transcription and ultimately inducing apoptosis [47,48,49,50]. Although this approach can facilitate mtDNA transfer from certain cell types, such as fibroblasts, optimization of ActD concentration is challenging. In our experience, residual ActD can persist in donor cells at levels sufficient to induce cytotoxicity in resulting cybrids and enucleation using an unoptimized ActD concentration can lead to complete failure to recover cybrids. Consequently, many laboratories continue to rely on physically enucleated cells or platelets as mtDNA donors, reflecting the practical limitations of ActD-based methods [33,34,35,36,51,52,53,54].

Alternative approaches for generating transmitochondrial cybrids have also been described, including MitoCeption, which introduces isolated mitochondria into recipient cells via centrifugation [55,56]; delivery using a photothermal nanoblade [57]; pressure-driven mitochondrial transfer [58,59]; and magnetomitotransfer, which employs anti-TOM22 magnetic beads to facilitate mitochondrial uptake [60]. However, these methods typically require prior isolation of mitochondria, a process that is labor-intensive, demands substantial starting material, and necessitates rapid downstream manipulation to preserve mitochondrial integrity [61,62,63,64,65]. These technical challenges, along with inconsistent reproducibility, have limited their widespread adoption.

Therefore, enucleated cells remain a practical and widely used source of mtDNA donors. In the present study, we describe and validate two methodological innovations to improve cybrid generation: chemical enucleation using mitomycin C (MMC) and selection of cybrids using mitochondrial uncouplers, including carbonyl cyanide m-chlorophenyl hydrazone (CCCP) and niclosamide. MMC was indicated as a potential agent for enucleation of donor cells by the long survival times, without proliferation, of treated fibroblast feeder cells [66]. Previous work in our lab has also demonstrated greater toxicity of mitochondrial uncouplers towards $\rho^{0}$ cells as compared to wild-type cells, making them a potential alternative cybrid selection agent to medium lacking uridine and pyruvate [67].

## 2. Materials and Methods

### 2.1. Cell Lines, Culture Conditions, and Reagents

MDA1 cells were generated in our laboratory by transducing the MDA-MB-231 breast cancer cell line with the retrovirus rv.2555, which encodes the red fluorescent protein mCherry and puromycin resistance. 143B$\rho^{0}$/4523#1 cells were generated by transducing 143B$\rho^{0}$ cells with the retrovirus rv.4523, which encodes EGFP. Transduced cells were sorted based on EGFP fluorescence, clonally plated, and the resulting clones were screened for stable EGFP expression. HeLa/2739 cells were generated by transducing HeLa cells with the retrovirus 2739, which encodes the reverse tetracycline-controlled transactivator (Tet-On Advanced system, Clontech, San Jose, CA, USA) and confers blasticidin resistance [68]. The 143B#6 cell line is a Mitochondrial Transcription Factor A (TFAM) GeneSwap derivative of the parental 143B cell line and carries a CRISPR-induced biallelic deletion of TFAM, along with retrovirus rv.4000 encoding TFAM cDNA and blasticidin resistance [69]. $\rho^{0}$ variants of 143B, HCT116, HT1080, and U2OS cells were generated in our laboratory as previously described [44,45,46] and were used as recipient cells. HeLa/2739 cells used as donor cells were treated with 10 μg/mL MMC and cryopreserved in liquid nitrogen for 10 years prior to use in this study.

Cells were cultured in Dulbecco’s Modified Eagle Medium (DMEM, VWR, Radnor, PA, USA) supplemented with 4.5 g/L glucose, 10% fetal bovine serum (FBS, Atlas Biologicals, Fort Collins, CO, USA), 50 μg/mL gentamicin (GoldBio, St. Louis, MO, USA), 50 μg/mL uridine (Fisher Scientific, Waltham, MA, USA), and 1 mM sodium pyruvate (hereafter referred to as +UP medium). Medium lacking uridine and pyruvate (−UP) contained all other components at the same concentrations. Cells were maintained at 37 °C in a humidified incubator (Fisher Scientific, Waltham, MA, USA) with 5%$CO_{2}$. No evidence of the reversion of the $\rho^{0}$ phenotype was observed for any of the $\rho^{0}$ cell lines despite extensive cultivation in the absence of mtDNA replication inhibitors and mtDNA-damaging agents.

All cell lines were authenticated by short tandem repeat (STR) analysis (LabCorp, Burlington, NC, USA) and were routinely screened for mycoplasma contamination.

### 2.2. Fusion Procedure

Donor cells were grown to confluency on a 150 mm plate, detached with 0.05% trypsin/0.53 mM EDTA (Fisher Scientific, Waltham, MA, USA), neutralized with 4 mL of complete medium, and 1 mL of the suspended cells was seeded on a new 150 mm plate and allowed to attach for 24 h. Media was then removed and replaced with +UP containing 10, 20, 40, or 80 μg/mLMMC (Fisher Scientific, Waltham, MA, USA) for 3 h. Simultaneously, ${1.5\times 10}^{6}$ recipient cells were seeded per well of a 6-well plate (3 wells total) and allowed to attach during the same 3 h period. Following MMC treatment, or directly from a thawed vial when utilizing frozen HeLa/2739 as donor cells, ${1.5\times 10}^{6}$ of the donor cells were added to each well containing recipient cells and allowed to attach for an additional 3 h. The medium was then removed, and cells were washed three times with DMEM lacking additives.

To induce donor–recipient fusion, each well was aspirated and media replaced with a solution of 45% polyethylene glycol with an average molecular weight of 1450 Da (PEG, Fisher Scientific, Waltham, MA, USA), 45% DMEM, and 10% dimethyl sulfoxide (DMSO, Fisher Scientific, Waltham, MA, USA). After 1.0 or 1.5 min, the PEG solution was removed, and cells were immediately washed three times with 10% DMSO in DMEM. Following the final wash, +UP medium was added, and cells were incubated overnight. The medium was replaced with fresh +UP the following morning, and cells were incubated for an additional 6 h. Cells were then detached, and either 10% or 30% of the total cell population was seeded onto 100 mm plates containing a selection medium.

### 2.3. Cybrid Selection

Selection was performed using either −UP medium or +UP medium containing mitochondrial uncouplers. Carbonyl cyanide m-chlorophenylhydrazone (CCCP, Fisher Scientific, Waltham, MA, USA) was used at concentrations of 0.5 μM or 1.0 μM, and niclosamide (Fisher Scientific, Waltham, MA, USA) was used at 0.25 μM. The selection medium was changed three times per week until colonies became visible to the naked eye (10–14 days post-fusion). Prior to colony picking, the perimeter of each colony was traced and filled in with a black permanent marker on the underside of the plate to record colony size and position for subsequent imaging and background analysis. Colonies were picked from the 100 mm plates using a 10 μL micropipette with a plastic tip and transferred individually to wells of a 96-well plate, with the pipette tip changed between each colony.

Starting concentrations of MMC, CCCP, and niclosamide were based on those reported in the literature and our own research [67], and were further refined in this study.

### 2.4. Cybrid Validation

Putative cybrids were validated for (1) the presence of mtDNA, (2) the origin of nDNA, and, where applicable, (3) the origin of mtDNA. The presence of mtDNA was confirmed by duplex PCR amplification of a 901 bp fragment from a mitochondrial hypervariable region and a 389 bp fragment from the nuclear 18S rRNA gene. The mtDNA fragment was amplified with the forward primer 5′-CAACCCGGTCAGCCCCTCTC-3′ and the reverse primer 5′-GCGGGGACGGGCGGTGGC-3′. The 18S rRNA region was amplified using the forward primer 5′-AATGTCTGCACAGCCACTTTCCAC-3′ and the reverse primer 5′-TCGTAGTGTTCTGGCGAGCAGTTT-3′. Primers were obtained from (Fisher Scientific, Waltham, MA). For the MDA1 (donor) and 143B$\rho^{0}$/4523#1 (recipient) cell line pair, mtDNA origin was determined by restriction fragment length polymorphism (RFLP) analysis of a 278 bp mtDNA fragment containing an NcoI restriction site in MDA1 but not in 143B cell lines. The fragment was amplified using the forward primer 5′-CAACCCGGTCAGCCCCTCTC-3′ and the reverse primer 5′-GCGGGGACGGGCGGTGGC-3′. A total of 1 μL of the PCR product was then added to 9 μL of rCutSmart Buffer and NcoI-HF (New England Biolabs, Ipswich, MA, USA) in water with a final enzyme concentration of 80 units/mL and incubated for 1 h at 37 °C. Products derived from MDA1 mtDNA were cleaved into 132 bp and 146 bp fragments, whereas products derived from 143B mtDNA remained undigested.

To verify the absence of a donor chromosome containing rv.4000 provirus, putative cybrids from the fusion of 143B#6 with HCT116$/4008\rho^{0}$ were tested for the presence of a provirus-encoded cDNA for human TFAM. To do so, a 349 bp region was amplified using the forward primer 5′-GTTGGAGGGAACTTCCTGATT-3′ and the reverse primer 5′-TGCTGAATATATAATTCCTTTTCAGAGT-3′.

### 2.5. Identification of the Nuclear Donor

Donor cell lines contained antibiotic resistance markers for either puromycin (MDA1) or blasticidin (HeLa/2739 and 143B#6), which were used to determine the origin of nDNA in cybrids. Both puromycin and blasticidin were sourced from Fisher Scientific, Waltham, MA. For each fusion experiment, two replicate 96-well plates were prepared: one containing +UP medium supplemented with either 3 μg/mL puromycin or 25 μg/mL blasticidin, and one containing +UP only. Wells A1 and B1 served as controls and were seeded with donor and recipient cells, respectively, while all remaining wells were seeded with putative cybrid clones. Cells were monitored for several days until all recipient control cells in antibiotic-containing wells appeared dead.

### 2.6. WST-8 Viability Assay

Cell viability in antibiotic-treated and untreated conditions was assessed using a water-soluble tetrazolium (WST-8, MedChemExpress, Monmouth Junction, NJ, USA) assay. A total of 100 μL of +UP medium containing 0.5 mM WST-8 and 20 μM 1-methoxy PMS (MedChemExpress, Monmouth Junction, NJ, USA) was added to each well. Absorbance at 450 nm was recorded on a VERSAmax microplate reader (Molecular Devices, San Jose, CA, USA) using SOFTmax PRO 4.3LS software at 0 h and 4 h to assess metabolic activity. Baseline-corrected absorbance values were used to generate a heat map in GraphPad Prism 8.4.2.

### 2.7. Clonogenic Assays

Cells were seeded into 12-well plates at densities of up to $7\times 10^{4}$ cells/well ($\rho^{0}$) or 150 cells/well ($\rho^{+}$) and allowed to attach overnight. Cells were then treated with varying concentrations of CCCP or niclosamide or left untreated as controls. Medium was replaced three times per week until colonies were visible in control wells.

### 2.8. Colony Staining, Imaging, and Analysis

Medium was aspirated from the plates, and cells were washed twice with phosphate-buffered saline (PBS). Colonies were stained with a crystal violet solution (0.5% crystal violet, 20% methanol (both Fisher Scientific, Waltham, MA, USA) in water) for 5 min, washed with deionized water, air-dried, and imaged with a BioRad (Hercules, CA, USA) ChemiDoc imaging system. Colony counts and background cell density were quantified using Fiji 2.17.0 [70].

### 2.9. Statistical Analysis

All statistical analyses were conducted using GraphPad Prism 8.4.2. A two-way analysis of variance (ANOVA) with Tukey’s post hoc was used to evaluate the effects of MMC concentration and selection medium type on cybrid colony counts. Background staining of surviving cells under different selection conditions was analyzed using either a log-transformed ANOVA with Tukey’s post hoc or a log-transformed unpaired *t*-test, as appropriate.

## 3. Results

### 3.1. $\rho^{0}$ Cells Are Sensitive to Mitochondrial Uncouplers Even at High Cell Densities

**Figure 1:**
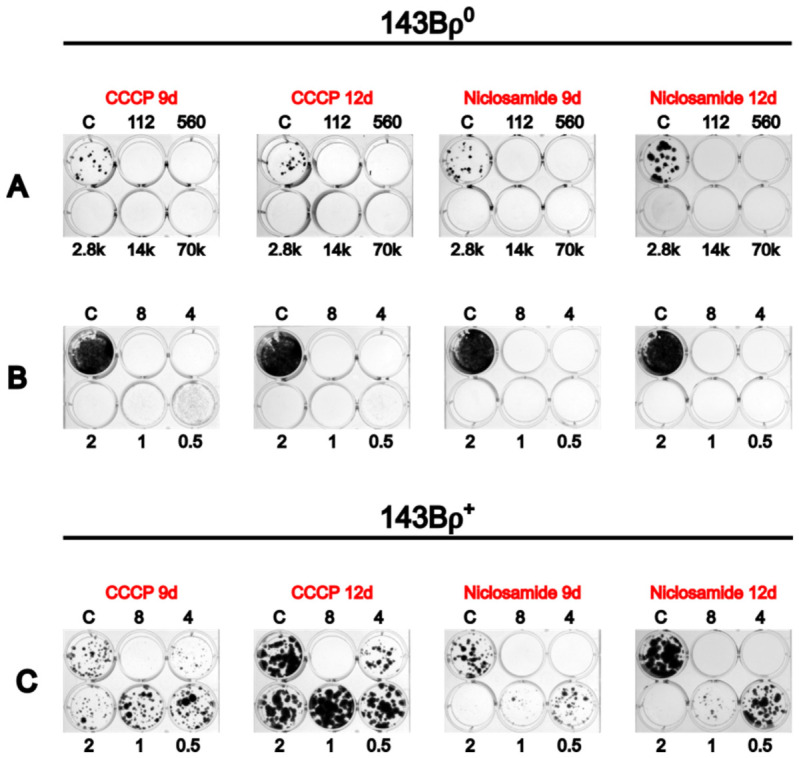
Mitochondrial uncouplers are more toxic to ρ^0^ than ρ^+^ cells at various cell densities. (**A**) Survival of 143Bρ^0^ cells in 2 μM CCCP or niclosamide when plated at different cell densities. The number of cells seeded per well is stated above or below the wells. Control wells were seeded with 112 cells per well and grown without uncouplers. (**B**) Dose responses of143Bρ^0^ cells to mitochondrial uncouplers when plated at 7 × 10^4^ cells per well. Concentrations of CCCP or niclosamide (in μM) are stated above or below wells. The number of days allowed for colony formation is indicated above each plate. (**C**) Dose responses of 143Bρ^+^ cells to CCCP and niclosamide when plated at 150 cells per well.

Previous work in our lab has demonstrated that mitochondrial uncouplers are more toxic to $\rho^{0}$ cells than to $\rho^{+}$ cells when plated at a low density of 150 cells per well of a 12-well plate [67]. However, this density of cells is lower than the density used for cybrid selection on 100 mm plates. In our experience, metabolic selection in −UP medium becomes less efficient at higher cell densities, likely due to the release of compensatory nutrients from dying cells. To determine whether cell density similarly affects selection with mitochondrial uncouplers, we assessed the sensitivity of 143B$\rho^{0}$ cells to 2 μM CCCP and 2 μM niclosamide across a range of cell densities. 143B$\rho^{0}$ cells were seeded on 12-well plates in 5-fold increments from 112 up to $7\times 10^{4}$ cells per well (Figure 1A). At these concentrations, both CCCP and niclosamide resulted in the death of 143B$\rho^{0}$ cells at all tested densities. Therefore, a follow-up experiment was conducted to identify optimal uncoupler concentrations for selection. In this experiment, duplicate 12-well plates were seeded with $7\times 10^{4}$ 143B$\rho^{0}$ cells per well, which is an equivalent density of $1.1\times 10^{6}$ cells seeded on a 100 mm plate (Figure 1B). Based on the expectation that wild-type cells would exhibit greater resistance to uncouplers and rapidly overgrow at high densities, an additional two 12-well plates were seeded with only 150 143B$\rho^{+}\;$cells per well (Figure 1C). All $\rho^{0}$ and $\rho^{+}$ cultures were then treated with serial dilutions of CCCP or niclosamide to determine concentrations that selectively eliminate $\rho^{0}$ cells while preserving $\rho^{+}\;$cell viability. After 12 days of treatment, 0.5 μM and 1.0 μM CCCP eliminated $\rho^{0}$ cells without significantly affecting $\rho^{+}$ cell growth (Figure 1B). At 2 μM and 4 μM of CCCP, as well as 0.5 μM and 1.0 μM of niclosamide, $\rho^{0}$ cells were eliminated, but the growth of $\rho^{+}$ cells was noticeably hindered. A total of 8 μM of CCCP and 2, 4, and 8 μM of niclosamide resulted in the elimination of both $\rho^{0}$ and $\rho^{+}$ cells after 12 days (Figure 1B,C).

### 3.2. MMC Is Capable of Producing Fusion-Competent Cytoplasts

Previously described methods of chemical enucleation have used ActD to inactivate nDNA [32]. Although chemical enucleation provides a more convenient alternative to physical enucleation, we have observed that enucleating concentrations of ActD can also arrest the growth of resulting cybrids. To address this limitation, we evaluated MMC as an alternative agent for enucleation of mtDNA donor cells. MMC has been shown to support prolonged survival of treated fibroblast feeder cells, consistent with effective inactivation of nDNA without immediate loss of viability [66]. This effect is mediated through the formation of covalent cross-links and single-point covalent adducts in nDNA by MMC [71].

**Figure 2:**
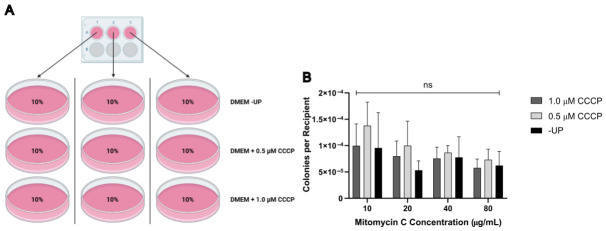
MMC enucleating concentration does not significantly affect cybrid colony yield. (**A**) Schematic representing the plating of post-fusion cells for a given MMC concentration. Created in BioRender. Weaver, L. (2026). (**B**) Statistical analysis of putative cybrid colonies per recipient produced from fusions with donors enucleated at different concentrations of MMC and selected with either CCCP or −UP. ns, not significant.

To assess the suitability of MMC for chemical enucleation, four replicate 150 mm plates of MDA1 donor cells (~80% confluent), which express the fluorescent protein mCherry and puromycin resistance, were treated with 10, 20, 40, or 80 μg/mL MMC for 3 h. Following treatment, $1.5\times 10^{6}$ donor cells were fused for 1 min with an equal number of eGFP-expressing, G418-resistant 143B$\rho^{0}$/4523#1 cells, as described in Materials and Methods. From each of the three wells in a 6-well plate, 10% of the cells were subjected to one of three selection conditions: (1) −UP, (2) +UP supplemented with 0.5 μM CCCP, or (3) +UP supplemented with 1.0 μM CCCP. Cells were cultured for 12 days with medium changes three times per week (Figure 2A). This design yielded nine 100 mm plates per fusion experiment for a total of 36 plates (four MMC concentrations × triplicate plates for each concentration × three selection modalities). After crystal violet staining, colonies were counted to evaluate the effects of MMC concentration and selection type on the yield of cybrid colonies. Although a decrease in colony number per recipient cell was observed with increasing concentrations of MMC, this trend did not reach statistical significance. Two-way analysis of variance revealed no significant effect of MMC concentration *F*(3,24) = 2.79, *p* = 0.06, selection medium *F*(2,24) = 1.95, *p* = 0.16, or their interaction *F*(6,24) = 0.29, *p* = 0.93, on colony count (Figure 2B). These results indicate that neither MMC concentration within the tested range, nor the use of CCCP instead of −UP, has a significant effect on the number of putative cybrid clones produced. Although we initially expected higher MMC concentrations to reduce colony counts, the absence of a significant effect may reflect the enzyme rate-limited activation of MMC by quinone reductases.

### 3.3. Selection with CCCP Can Reduce Background

**Figure 3:**
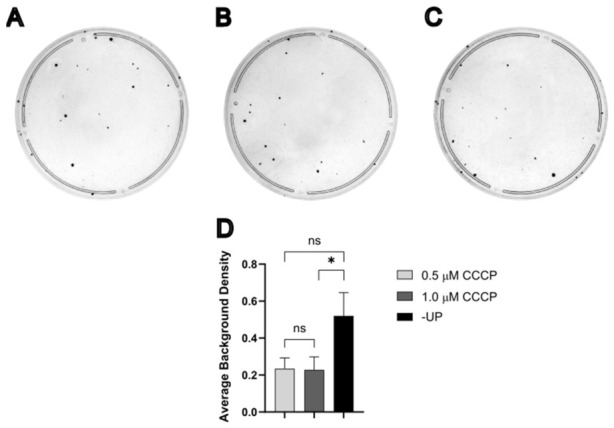
Selection with CCCP reduces average background staining. (**A**–**C**) Representative plates of the 1.0 min fusion between MDA1 (donor) and 143Bρ^0^/4523#1 (recipient) after selection with 0.5 μM CCCP (**A**), 1.0 μM CCCP (**B**), or −UP (**C**). (**D**) Statistical analysis of background staining all plates from (**A**–**C**) in arbitrary units (*n* = 12 per selection method). *, *p* < 0.05. ns, not significant.

Plates generated from MDA1 × 143B$\rho^{0}$/4523#1 fusion experiments were analyzed to quantify background staining arising from cells not associated with discrete colonies (Figure 3A–C). Background cell density was estimated by measuring the average pixel intensity of residual crystal violet staining after excluding colonies and is reported in arbitrary units. This residual staining is presumed to reflect surviving, without considerable proliferation, donor and/or recipient cells. The distribution of background intensity values was assessed using the Anderson–Darling test, which indicated that the data were better described by a lognormal distribution than a Gaussian distribution. Accordingly, statistical analysis was performed using a log-transformed analysis of variance. This analysis revealed a significant effect of the selection medium on background staining, $F\left(2,33\right)=3.87$, p=0.03. Post hoc comparisons using Tukey’s Honestly Significant Difference test showed no significant difference in mean background staining between 0.5 μM CCCP and either −UP (*p* = 0.09) or 1.0 μM CCCP (*p* = 0.91). However, background staining was significantly higher in the −UP medium compared to 1.0 μM CCCP (*p* = 0.04) (Figure 3D). Although these differences are not readily apparent by visual inspection, this analysis indicates that selection with 1.0 μM CCCP is most effective at reducing background cell density surrounding cybrid colonies.

### 3.4. Fusion Time Affects the Yield of Cybrids

**Figure 4:**
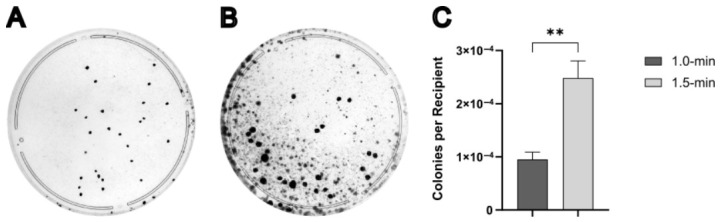
Fusion time affects cybrid yield. (**A**,**B**) Representative plates of the fusions between MDA1 (donor) and 143Bρ^0^/4523#1 (recipient) after exposure to PEG for 1.0 min (**A**) or 1.5 min (**B**). (**C**) Statistical analysis of putative cybrid colonies per recipient counted on all plates from (**A**,**B**). **, *p* < 0.01.

Standard protocols typically recommend a 1.0 min fusion duration for cybrid generation [31,32]. To evaluate the impact of fusion time on cybrid yield, MDA1 × 143B$\rho^{0}$/4523#1 fusions were performed using donor cells enucleated with 10 μg/mL MMC and fusion times of 1.0-, 1.5-, or 2.0 min. Following fusion, 30% of the cells were plated onto three 100 mm plates containing −UP for selection. After picking clones, plates were stained with a crystal violet solution, and colonies were counted as described in Section 2 Materials and Methods. Colony counts were successfully obtained for the 1.0 and 1.5 min fusion conditions, but the 2.0 min fusions exhibited excessive overgrowth, precluding accurate colony quantification (Figure 4A,B). A log-transformed unpaired *t*-test revealed a significant increase in colony number per recipient when fusion time was extended from 1.0 min to 1.5 min, $t\left(4\right)=7.58$, p=0.002 (Figure 4C). Despite this increase in yield, plates from the 1.5 min fusion showed substantially elevated background cell density. This limitation prompted further evaluation of alternative selection methods before adopting 1.5 min fusion times for subsequent experiments.

### 3.5. CCCP Selection Reduces Background Staining at Elevated Plating Densities

**Figure 5:**
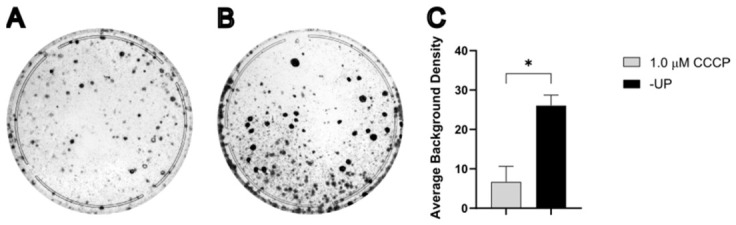
Selection with CCCP reduces background. (**A**,**B**) Representative plates of the 1.5 min fusion between MDA1 (donor) and 143Bρ^0^/4523#1 (recipient) after selection with 1.0 μM CCCP (**A**) or −UP (**B**). (**C**) Statistical analysis of background staining of all plates from (**A**,**B**) in arbitrary units (*n* = 3 per selection method). *, *p* < 0.05.

To further validate the efficacy of CCCP for cybrid selection, we compared the CCCP selection with −UP under conditions of increased fusion time and plating density. MDA1 donor cells were enucleated with 10 μg/mL of MMC, fused with 143B$\rho^{0}$/4523#1 recipient cells for 1.5 min, and 30% of the post-fusion cells were plated onto three 100 mm plates containing 1.0 μM CCCP (Figure 5A). Background cell density was quantified as described in the previous experiment and compared to that observed in 1.5 min fusion plates selected with the −UP medium (Figure 5B). Statistical analysis using an unpaired *t*-test on log-transformed data demonstrated a significant reduction in background staining in CCCP-selected plates relative to −UP controls, 1.0 μM CCCP, $t\left(4\right)=4.25$, p=0.01 (Figure 5C). The difference in background density between −UP and 1.0 μM CCCP plates was more readily apparent under these higher-density plating conditions (30% of post-fusion cells) than in the previous experiment in which only 10% of cells were plated. This reduction in background staining facilitates easier colony identification and allows fewer replicate plates to be used to obtain an optimal number of cybrid clones. Based on these results, subsequent experiments were performed using a 1.5 min fusion duration, plating 30% of post-fusion cells, and selecting with mitochondrial uncouplers.

### 3.6. Putative Cybrid Clones Contain Donor-Derived mtDNA

**Figure 6:**
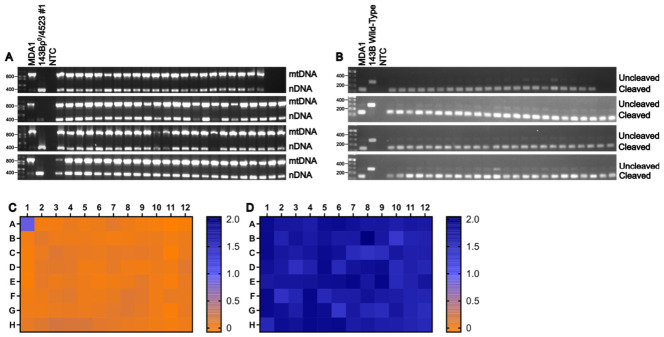
MDA1 × 143Bρ^0^/4523#1 clones are true cybrids. (**A**) Putative cybrids contain mtDNA. (**B**) mtDNA in putative cybrids originates from MDA1 cells. NTC, no-template control. (**C**,**D**) Heat maps of WST-8 assay absorbance values at 450 nm of two replica plates grown either with (**C**) or without (**D**) 3 μg/mL puromycin.

The presence of mtDNA in putative cybrid clones was confirmed by duplex amplification of nDNA and mtDNA regions, as described in Materials and Methods (Figure 6A). This $\rho^{0}$ test confirmed the presence of mtDNA in all selected clones. To authenticate MDA1 as the source of mtDNA in the putative cybrids, we used the C/T single-nucleotide polymorphism (SNP) at mt16366, which affects the NcoI site (present in MDA1, absent in 143B). A 278 bp mtDNA fragment encompassing this restriction site was amplified from putative cybrids, digested with NcoI for 1 h, and analyzed by gel electrophoresis. Fragments derived from MDA1 mtDNA were cleaved into 132 bp and 146 bp products (which co-migrated as a single band due to similar size), whereas fragments derived from 143B mtDNA remained intact. As anticipated, this RFLP analysis demonstrated that all putative cybrid clones harbored mtDNA originating from MDA1 (Figure 6B).

### 3.7. Putative Cybrids Lack Donor-Derived Provirus-Carrying Chromosomes

MDA1 donor cells are puromycin-resistant, whereas the 143B$\rho^{0}$/4523#1 recipient cells are puromycin-sensitive. To determine whether putative cybrids retained donor-derived chromosomes carrying the provirus present in MDA1, clones were cultured in the presence or absence of 3 μg/mL puromycin in duplicate plates. Survival in the presence of puromycin would indicate either persistence of MMC-treated donor cells or transfer and maintenance of provirus-containing donor chromosomes. Putative cybrids analyzed in this assay were derived from fusions in which MDA1 cells were enucleated with 10 μg/mL MMC, fused for 1.5 min with 143B$\rho^{0}$/4523#1, and selected in −UP medium. Cell viability was assessed using a water-soluble tetrazolium (WST-8) assay, with absorbance measured at 450 nm. All putative cybrid clones, as well as the 143B$\rho^{0}$/4523#1 control (well B1), failed to survive the puromycin treatment, whereas MDA1 control cells (well A1) remained viable (Figure 6C,D). These results indicate that the cybrid clones did not retain donor-derived chromosomes carrying the provirus rv.2555.

143B$\rho^{0}$ osteosarcoma cells are the most commonly used recipients for cybrid formation; however, the use of cell lines derived from other tissues is often needed for biological relevance. Previous work in our lab has also demonstrated that different $\rho^{0}\;$cell lines exhibit variable sensitivity to both −UP selection and mitochondrial uncouplers [67]. Therefore, we evaluated the feasibility of using HCT116/4008$\rho^{0}\;(colorectal\;carcinoma)$, HT1080$\rho^{0}\;(fibrosarcoma)$, and U2OS$\rho^{0}$ (osteosarcoma), all of which exhibit epithelial morphology.

### 3.8. HCT116$\rho^{0}$ Cells Are Useful Recipients When Selected with CCCP

**Figure 7:**
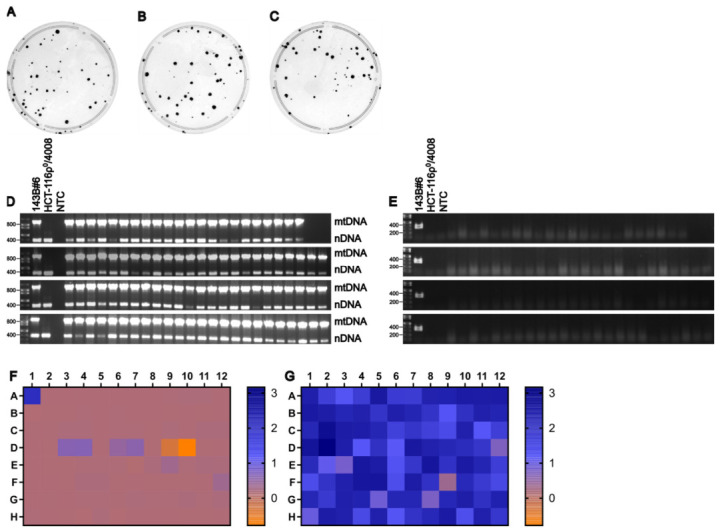
HCT116/4008ρ^0^ is a useful recipient cell line. (**A**–**C**) Plates from three biological replicates of the fusion between 143B#6 cells (donor) and HCT116/4008ρ^0^ (recipient). (**D**) Duplex PCR test for the presence of mtDNA in putative cybrids. (**E**) PCR test for the presence of an hTFAM-encoding region of cDNA. NTC, no-template control. (**F**,**G**) Heat maps of WST-8 assay absorbance values at 450 nm of two replica plates grown either with (**F**) or without (**G**) 25 μg/mL blasticidin.

To assess the suitability of HCT116${/4008\rho }^{0}$ cells as recipients, ~80% confluent 150 mm plates of 143B#6 donor cells were enucleated with 10 μg/mL of MMC for 3 h. $1.5\times 10^{6}$ of the resulting cytoplasts were fused for 1.5 min with an equal number of HCT116$/4008\rho^{0}$ recipient cells, as described in Materials and Methods. Following fusion, 30% of the cells from each of three replicate wells were plated onto 100 mm plates and selected using +UP medium supplemented with 1.0 μM CCCP. After colonies were picked, plates were stained with crystal violet solution to visualize colony formation and background staining (Figure 7A–C). Consistent with the previous experiment, mtDNA was detected in all clones (Figure 7D). The absence of donor-derived chromosomes carrying the provirus 4000 was initially assessed by culturing clones in the presence or absence of 25 μg/mL blasticidin, followed by a WST-8 assay (Figure 7E). This assay yielded inconclusive results, as some clones exhibited low but detectable metabolic activity compared to the 143B#6 control. To more definitively assess the presence of donor-derived nuclear material, PCR amplification targeting a 349 bp region of hTFAM cDNA (present in 143B#6 donor cells but absent in HCT116${/4008\rho }^{0}$ recipient cells) was performed (Figure 7F). No amplification products were detected in any cybrid clones, indicating the absence of the provirus 4000 and confirming that nDNA originated from the recipient cells. Together, the $\rho^{0}$ test, +/− blasticidin WST-8 assay, and hTFAM PCR analysis demonstrate that each of the 94 clones selected were true transmitochondrial cybrids, demonstrating that HCT116$/4008\rho^{0}$ cells are a viable recipient line for cybrid generation.

### 3.9. HT1080$\rho^{0}$ Cells Can Be Used as Recipients When Selecting with Niclosamide

**Figure 8:**
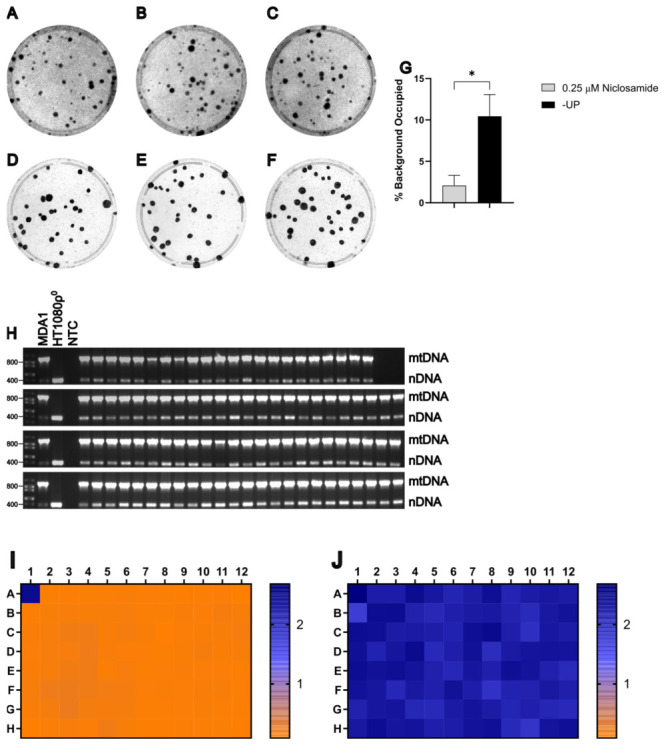
HT1080ρ^0^ is a viable recipient cell line. (**A**–**F**) Plates from three biological replicates of the fusion between MDA1 (donor) and HT1080ρ^0^ (recipient) selected with either −UP (**A**–**C**) or 0.25 μM niclosamide (**D**–**F**). (**G**) Statistical analysis of background staining of (**A**–**F**) in arbitrary units (*n* = 3 per selection method). *, *p* < 0.05. (**H**) Duplex PCR test for the presence of mtDNA in putative cybrids. NTC, no template control. (**I**,**J**) Heat maps of WST-8 assay absorbance values at 450 nm of two replica plates grown either with (**I**) or without (**J**) 3 μg/mL puromycin.

To evaluate whether HT1080 $\rho^{0}$ cells are useful as recipients when selecting with niclosamide, MDA1 donor cells were grown to ~80% confluence on 150 mm plates and enucleated with 10 μg/mL of MMC for 3 h. $1.5\times 10^{6}$ MDA1 cytoplasts were fused for 1.5 min with an equal number of HT1080$\rho^{0}$ recipient cells, as described in Materials and Methods. Previous work in our lab indicates that HT1080$\rho^{0}$ cells exhibit greater resistance to CCCP compared to other recipient cell lines. Therefore, 30% of the cells from each replicate well were plated onto 100 mm plates and selected using either +UP medium supplemented with 0.25 μM niclosamide or −UP medium. A total of 94 clones selected with 0.25 μM niclosamide were picked and plates were stained with crystal violet to visualize colonies and assess background staining (Figure 8A–F). Statistical analysis using an unpaired *t*-test with log-transformed data demonstrated that selection with 0.25 μM niclosamide significantly reduced background staining compared to −UP selection, $t\left(4\right)=4.13$, p=0.01 (Figure 8G). Consistent with previous experiments, mtDNA was detected in all niclosamide-selected clones (Figure 8H). The absence of donor-derived chromosomes carrying the provirus rv.2555 was evaluated by culturing clones in the presence or absence of 3 μg/mL puromycin, followed by a WST-8 assay (Figure 8I,J). The $\rho^{0}$ test and +/− puromycin WST-8 assay indicate that each of the 94 clones selected was likely a true transmitochondrial cybrid, demonstrating that HT1080$\rho^{0}$ cells are a viable candidate recipient cell line for cybrid generation.

### 3.10. U2OS$\rho^{0}$ Cells Can Be Used as Recipients When Selecting with Niclosamide

**Figure 9:**
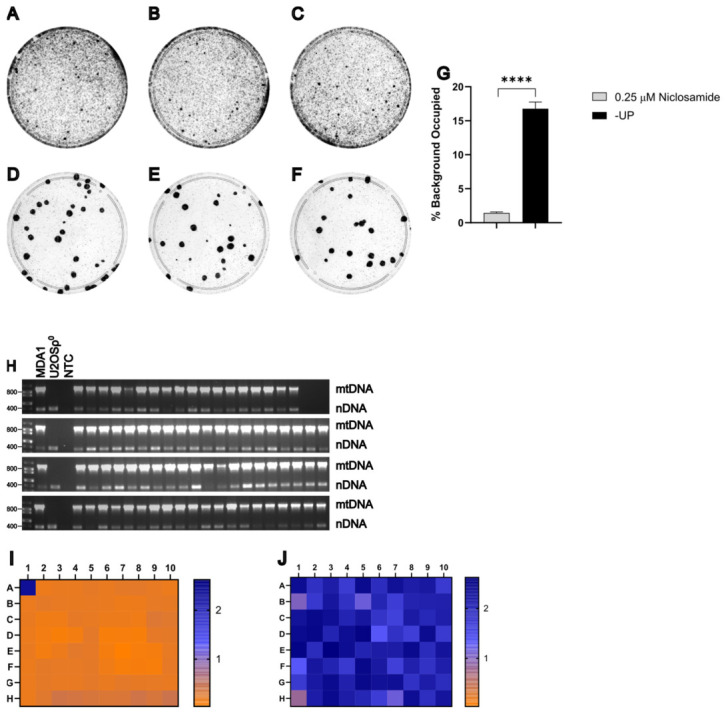
U2OS$\rho^{0}$ is a viable candidate recipient cell line. (**A**–**F**) Plates from three biological replicates of the fusion between MDA1 (donor) and U2OS$\rho^{0}$ (recipient) selected with either −UP (**A**–**C**) or 0.25 μM niclosamide (**D**–**F**). (**G**) Statistical analysis of background staining of (**A**–**F**) (*n* = 3 per selection method). ****, p<0.0001. (**H**) Duplex PCR test for the presence of mtDNA in putative cybrids. NTC, no template control. (**I**,**J**) Heat maps of WST-8 assay absorbance values at 450 nm of two replica plates grown either with (**I**) or without (**J**) 3 μg/mL puromycin.

MDA1 donor cells were grown to ~80% confluence on 150 mm plates and enucleated with 10 μg/mL of MMC for 3 h. MDA1 cytoplasts of $1.5\times 10^{6}$ were fused for 1.5 min with U2OS$\rho^{0}$ recipient cells, as described in Materials and Methods. Previous work in our lab indicates that U2OS$\rho^{0}$ cells exhibit greater resistance to CCCP than other recipient cell lines tested. Therefore, 30% of the cells from each replicate well were plated onto 100 mm plates and selected using either +UP medium supplemented with 0.25 μM niclosamide or −UP medium. This fusion yielded fewer putative cybrids than previous experiments; therefore, 78 clones selected with 0.25 μM niclosamide were picked. Plates were stained with crystal violet to visualize colonies and assess background staining (Figure 9A–F). Statistical analysis using an unpaired *t*-test with log-transformed data demonstrated that selection with 0.25 μM niclosamide significantly reduced background staining compared to −UP selection, $t\left(4\right)=35.95$, p<0.0001 (Figure 9G). Consistent with previous experiments, mtDNA was detected in all clones (Figure 9H). The absence of donor-derived chromosomes carrying the provirus rv.2555 was assessed by culturing clones in the presence or absence of 3 μg/mL puromycin, followed by a WST-8 assay (Figure 9I,J). The $\rho^{0}$ test and +/− puromycin WST-8 assay indicate that each of the 78 clones selected was likely a true transmitochondrial cybrid, demonstrating that U2OS$\rho^{0}$ cells are a viable recipient line for cybrid generation.

### 3.11. MMC-Inactivated Donor Cells Retain Fusion Competence for Many Years

The ability to freeze MMC-inactivated donor cells provides flexibility and allows for experimental pause points in the event of procedural errors during fusion. In addition, MMC is unstable in solution and loses potency over time; therefore, storing enucleated cells in liquid nitrogen enables the preparation of large batches of donor cytoplasts while MMC is most potent. These cytoplasts can later be thawed and directly used for fusion with recipient cells as needed.

**Figure 10:**
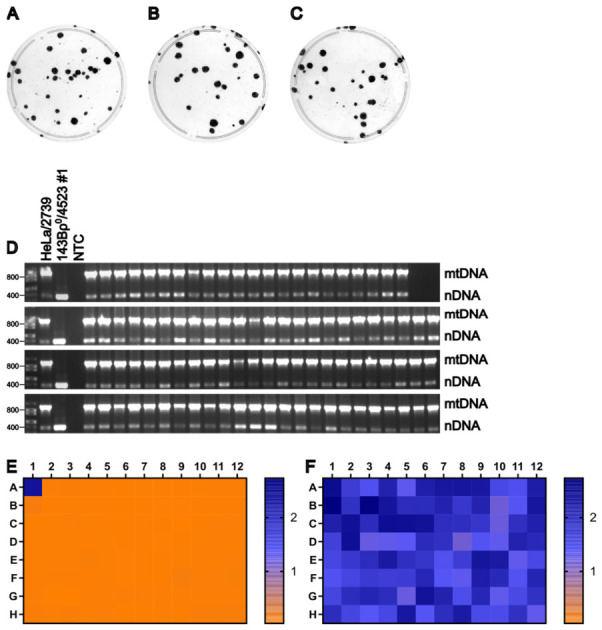
MMC-inactivated donor cells retain fusion competence for extended periods. (**A**–**C**) Plates from three biological replicates of the fusion between HeLa/2739 (donor) and 143B$\rho^{0}$/4523#1 (recipient). (**D**) Duplex PCR test for the presence of mtDNA in putative cybrids. NTC, no-template control. (**E**,**F**) Heat map of WST-8 assay absorbance values at 450 nm of two replica plates grown either with (**E**) or without (**F**) 25 μg/mL blasticidin.

Blasticidin-resistant HeLa/2739 donor cells were grown to ~80% confluence on 150 mm plates and enucleated with 10 μg/mL of MMC for 3 h. The resulting cytoplasts were cryopreserved in liquid nitrogen for 10 years prior to fusion. Frozen HeLa/2739 cytoplasts were thawed and $1.5\times 10^{6}$ cells were plated into each of three wells of a 6-well plate. After a 3 h attachment period, $1.5\times 10^{6}$ 143B$\rho^{0}$/4523#1 cells were added to each well containing cytoplasts and allowed to attach for an additional 3 h. The fusion was then performed for 1.5 min, as described in Materials and Methods. Following fusion, 30% of the cells from each of the three replicate wells were plated on 100 mm plates and selected using +UP medium supplemented with 1.0 μM CCCP. Colonies were picked and plates stained with crystal violet to visualize colonies and assess background staining (Figure 10A–C). Consistent with previous experiments, mtDNA was detected in all clones (Figure 10D). The absence of donor-derived chromosomes containing the provirus 2739 was assessed by culturing clones in the presence or absence of 25 μg/mL blasticidin, followed by a WST-8 assay (Figure 10E,F). The $\rho^{0}$ test and +/− blasticidin WST-8 assay indicate that each of the 94 clones selected was likely a true transmitochondrial cybrid. These results confirm that MMC-inactivated donor cells retain fusion competency even after long-term storage in liquid nitrogen.

## 4. Discussion

Here, we present an alternative method of chemical enucleation using MMC, an antitumor antibiotic typically used in vitro to arrest fibroblasts in the G2/M phase as feeder cells in stem cell cultures. MMC inactivates DNA through the formation of covalent interstrand and intrastrand cross-links, as well as single-point covalent adducts. This activity is enzyme rate-limited and requires reduction in the quinone moiety by cellular quinone reductases [71]. As a result, MMC-treated cells are functionally enucleated without triggering immediate apoptosis. In our experience, a fraction of MMC-treated cells can survive for up to a month without proliferation. We also report that varying MMC concentration and treatment duration did not adversely affect fusion efficiency, likely reflecting the enzyme rate-limited mechanism of MMC. Analysis of clones generated using MMC-enucleated donor cells confirmed the presence of mtDNA and the absence of provirus-containing chromosomes, indicating successful generation of true transmitochondrial cybrids. Based on these findings, we propose MMC treatment as a method for chemical enucleation of mtDNA donor cells prior to fusion with $\rho^{0}$ recipient cells for cybrid generation.

A medium lacking uridine and pyruvate (−UP) is typically used for selection against $\rho^{0}$ cells following fusion [31,32,72]. Here, we describe an alternative selection strategy by utilizing the mitochondrial uncoupler CCCP or the anthelmintic niclosamide, both of which have been shown to selectively eliminate $\rho^{0}$ cells [67]. CCCP and niclosamide function as protonophores that dissipate the MMP by shuttling protons through the IMM into the matrix, thereby uncoupling the proton pumping from ATP synthase activity [73,74,75,76,77]. Although $\rho^{0}$ cells lack a functional respiratory chain, they maintain a mitochondrial membrane potential by hydrolyzing glycolytically derived ATP [78,79,80,81]. Due to their OXPHOS deficiency, these cells have a reduced capacity for ATP production and are therefore especially sensitive to mitochondrial uncoupling. Exposure to CCCP or niclosamide induces futile cycles of ATP generation and hydrolysis via reverse ATP synthase activity, ultimately leading to cell death [67]. We found that selection with uncouplers produced no significant difference in cybrid colony yield per recipient compared to −UP selection, indicating no additional negative effect on cybrid growth. Additionally, uncoupler selection reduced background cell density on post-fusion plates, facilitating easier colony picking, particularly at high plating densities.

We also observed that increased fusion time increases both cybrid yield and background, which indicates that this parameter can be used to optimize fusions. In practical terms, the choice of PEG exposure would depend on the specific experimental objective. When maximizing cybrid recovery is the priority (e.g., when using recipient cell lines known or expected to yield fewer cybrids), a longer fusion time may be justified despite the accompanying increase in background. Conversely, when cybrid yield is sufficient, a shorter fusion time is preferable because it provides a cleaner background and facilitates colony identification and isolation.

Across the study, cybrid yields varied approximately 5-fold between fusion modes (5.33 × 10^−5^ ± 1.76 × 10^−5^ to 3.04 × 10^−4^ ± 5.25 × 10^−5^), with most values clustering around 1 × 10^−4^ (Table 1). These yields are consistent with those reported for other methods. Thus, our approach offers greater robustness and simplicity but does not necessarily increase efficiency, which may be limited by the inherent constraints of PEG-mediated fusion.

## 5. Conclusions

We believe that, collectively, our findings indicate that MMC could be a superior chemical enucleating agent as compared to ActD, and that selection of cybrids using uncouplers is not only feasible but, in some cases, may be preferable to selection in a medium lacking uridine and pyruvate. A combined selection with uncouplers in the media devoid of uridine and pyruvate is worth further exploration in terms of reducing background, especially in experiments utilizing increased fusion times.

## Figures and Tables

**Table 1:** Cybrid yields per recipient cell across the study.

Donor	Recipient	MMC (µg/mL)	Fusion Time (min)	Selection	Cybrid Yield ± SD
MDA1	143Bρ^0^/4523#1	10	1.0	−UP	9.56 × 10^−5^ ± 6.71 × 10^−5^
MDA1	143Bρ^0^/4523#1	10	1.0	0.5 µM CCCP	1.38 × 10^−4^ ± 4.44 × 10^−5^
MDA1	143Bρ^0^/4523#1	10	1.0	1.0 µM CCCP	1 × 00^−4^ ± 4.16 × 10^−5^
MDA1	143Bρ^0^/4523#1	20	1.0	−UP	5.33 × 10^−5^ ± 1.76 × 10^−5^
MDA1	143Bρ^0^/4523#1	20	1.0	0.5 µM CCCP	1 × 00^−4^ ± 4.67 × 10^−5^
MDA1	143Bρ^0^/4523#1	20	1.0	1.0 µM CCCP	8 × 00^−5^ ± 2.91 × 10^−5^
MDA1	143Bρ^0^/4523#1	40	1.0	−UP	7.78^−5^ ± 3.91 × 10^−5^
MDA1	143Bρ^0^/4523#1	40	1.0	0.5 µM CCCP	8.67 × 10^−5^ ± 1.33 × 10^−5^
MDA1	143Bρ^0^/4523#1	40	1.0	1.0 µM CCCP	7.56 × 10^−5^ ± 2.14 × 10^−5^
MDA1	143Bρ^0^/4523#1	80	1.0	−UP	6.22 × 10^−5^ ± 2.69 × 10^−5^
MDA1	143Bρ^0^/4523#1	80	1.0	0.5 µM CCCP	7.33 × 10^−5^ ± 2.0 × 10^−5^
MDA1	143Bρ^0^/4523#1	80	1.0	1.0 µM CCCP	5.78 × 10^−5^ ± 1.68 × 10^−5^
MDA1	143Bρ^0^/4523#1	10	1.0	−UP	9.48 × 10^−5^ ± 1.36 × 10^−5^
MDA1	143Bρ^0^/4523#1	10	1.5	−UP	2.48 × 10^−4^ ± 3.22 × 10^−5^
MDA1	143Bρ^0^/4523#1	10	1.5	1.0 µM CCCP	1.75 × 10^−4^ ± 8.24 × 10^−5^
MDA1	143Bρ^0^/4523#1	10	2.0	−UP	TMTC ^1^
143B#6	HCT116/4008ρ^0^	10	1.5	1.0 µM CCCP	1.42 × 10^−4^ ± 3.85 × 10^−6^
MDA1	HT1080ρ^0^	10	1.5	−UP	1.25 × 10^−4^ ± 2.06 × 10^−5^
MDA1	HT1080ρ^0^	10	1.5	0.25 µM nicl.	8.07 × 10^−5^ ± 1.28 × 10^−6^
MDA1	U2OSρ^0^	10	1.5	−UP	3.04 × 10^−4^ ± 5.25 × 10^−5^
MDA1	U2OSρ^0^	10	1.5	0.25 µM nicl.	6.59 × 10^−5^ ± 1.56 × 10^−5^
HeLa/2739	143Bρ^0^/4523#1	10	1.5	1.0 µM CCCP	8.15 × 10^−5^ ± 8.41 × 10^−6^

^1^ TMTC, too many to count.

## Data Availability

The data presented in this study are contained in the article.

## Abbreviations

The following abbreviations are used in this manuscript:

MMC	Mitomycin C
CCCP	carbonyl cyanide m-chlorophenylhydrazone
DMEM	Dulbecco’s Modified Eagle Medium
IMM	Inner Mitochondrial Membrane
ATP	Adenosine Triphosphate
MMP	Mitochondrial Inner Membrane Potential
OXPHOS	Oxidative Phosphorylation
mtDNA	Mitochondrial DNA
nDNA	Nuclear DNA
−UP	Complete DMEM medium lacking uridine and pyruvate
+UP	Complete DMEM medium containing uridine and pyruvate
ActD	Actinomycin D
tRNAs	Transfer RNAs
rRNAs	Ribosomal RNAs
DMSO	Dimethyl Sulfoxide
STR	Short Tandem Repeats
PEG	Polyethylene Glycol
EDTA	Ethylenediaminetetraacetic Acid
EGFP	Enhanced Green Fluorescent Protein
